# Sex Differences at Early Old Stage in Glycolipid Metabolism and Fatty Liver in Offspring Prenatally Exposed to Chinese Great Famine

**DOI:** 10.3389/fnut.2022.913966

**Published:** 2022-06-22

**Authors:** Yumeng Zhang, Jianhong Pu, Yi Ding, Lei Wu, Yongxiang Yin, Mingya Sun, Ying Gu, Daiyi Zhang, Ze Zhang, Qiutong Zheng, Qinyuan He, Ting Xu, Yun He, Hongyu Su, Xiuwen Zhou, Lingjun Li, Yang Ye, Jingyang Li, Zhice Xu

**Affiliations:** ^1^Institute for Fetology, The First Affiliated Hospital of Soochow University, Suzhou, China; ^2^The Center of Management, The First Affiliated Hospital of Soochow University, Suzhou, China; ^3^Department of Preventive Medicine, College of Clinical Medicine, Suzhou Vocational Health College, Suzhou, China; ^4^Suzhou Industrial Park Centers for Disease Control and Prevention, Suzhou, China; ^5^Wuxi Maternity and Child Health Care Hospital, Wuxi, China

**Keywords:** famine, glycolipd metabolism, sex differences, catch-up growth, aging offspring

## Abstract

**Background:**

About 50 years ago, Chinese Great Famine (CGF) affected the entire population in China, and its long-term influence on the offspring has attracted significant attention for research. However, information on possible metabolic differences between sexes is limited. This study explored whether there might be sex differences in the risks of development of glucolipid metabolic dysfunction and fatty liver following prenatal exposure to CGF.

**Materials and Methods:**

There were 11,417 subjects around 55 years of age (6,661 women and 4,756 men). They were divided as the exposed group in which the fetal stage was in CGF, and the unexposed group included those born after CGF. Analysis focused on comparisons between sexes.

**Results:**

Compared to the unexposed group, the BMI and triglyceride (*P* < 0.05) in men were higher in exposed group, while waist circumference and blood sugar (*P* < 0.05) in the exposed women were significantly higher. With the ages being properly balanced, the risks of glycolipid metabolic dysfunction were significantly higher in both men and women in the exposed than in the unexposed group (*P* < 0.001). Prenatal exposure to CGF significantly increased risks of abnormal BMI (*P* < 0.001, 95% CI: 2.305–2.93), blood sugar (*P* < 0.05, 95% CI: 1.050–1.401), triglycerides (*P* < 0.05, 95% CI: 1.006–1.245), and fatty liver (*P* < 0.001, 95% CI: 1.121–1.390) in men, and increased risks of abnormal blood sugar (*P* < 0.05, 95% CI: 1.024–1.689) and positive urine sugar (*P* < 0.05, 95% CI: 1.062–6.211) in women. Height and body weight were either the same or higher in the exposed subjects compared with the unexposed ones, regardless of sexes.

**Conclusion:**

This study is the first to identify sex differences in the long-term effects of CGF on metabolism and fatty liver. Importance of the findings include the benefits of prescribing medicine for the early prevention of certain diseases for each sex before aging based on the differences revealed. This study also shows “catch-up growth” in the offspring prenatally exposed to CGF as possible mechanisms underlying the long-term effects.

## Introduction

Progress has been significantly made in the past 35 years in demonstration of adult diseases in fetal origins with the Barker’s theory; increasing evidence has shown that nutritional status in early life has a long-term impact on health or diseases in later life (1). Malnutrition during pregnancy could cause metabolic and molecular changes that may increase the risks of diseases in the offspring (2–4). Multiple studies have demonstrated that exposure to famine during fetal stages may increase risks of the development of obesity, hypertension, diabetes, and other diseases in adulthood (5–10). Notably, Chinese Great Famine (CGF), also called “Three Year of Hungry” (1959–1961), has become a special research model for the study of long-term influence of malnutrition during pregnancy on the offspring following “Dutch hungry” model in Europe (11–13). CGF occurred 50 years ago when China terribly lacked medical conditions; birth records at that time cannot be usually obtained at present. However, CGF affected whole country and whole populations. One report estimated that about 30 million people died due to food shortage (14) and CGF was the largest famine in human history. This tragedy also left a heritage for human being with a large population of male and female offspring who are at least 50 years of age (early stage of old) now, for investigations of long-term influence of malnutrition in early life periods. Although several studies reported the effects of CGF on cardiovascular, metabolic, and mental diseases in the offspring (5–10), there has been very limited information on CGF’s influence on possible metabolic differences between sexes.

A number of studies have shown that same adverse causes can induce different consequences between sexes. For instance, a previous study demonstrated that women are more susceptible than men to autoimmune diseases (15), and there were differences between men and women when they face the same intrauterine malnutrition (16). Whether CGF may also affect the male and female offspring at an early stage of old differently in metabolic regulations and outcomes is largely unknown. Previous studies have shown that famine during pregnancy increased the risk of abnormal glucose and lipid metabolism in the offspring (7, 8, 10). Most of those studies did not pay attention on possible sex differences. However, more and more studies have demonstrated that the occurrence and development of some diseases would be different due to sex difference (16–18). Therefore, this study was conducted to explore whether exposure to CGF during fetal stages may have sex differences in glucolipid metabolic dysfunction in the offspring.

Since the Barker’s theory was introduced three decades ago, multiple hypotheses have been proposed in explanation of possible mechanisms underlying adult diseases in fetal origins. Among them, “catch-up growth” is well known in interpreting why poor nutrition during pregnancy could cause adult diseases in later life (19, 20). Briefly, fetal growth restriction and low birth weight (LBW) would face a possible catch-up growth later, which may underlie the pathophysiological process of diseases. Although it is impossible now to obtain birth records during CGF, it should be certain that LBW did widely occur during CGF because of a large amount of data. First, a large number of epidemic and laboratory studies have confirmed that poor nutrition or famine during pregnancy can definitely produce LBW babies (13, 21, 22). Second, CGF caused poor nutrition and hungry, which was much serious than that of Dutch-hungry, because millions died due to food shortage (14). Thus, LBW was inevitable. This gave us an idea that certain physiological values of the offspring at an early stage of old, including their weight, height, and BMI, could be obtained. These data provided us opportunities to determine whether there might be a “catch-up growth” for the offspring prenatally exposed to CGF, and analysis how catch-up growth was linked to possible metabolic changes in both sexes, as the first time in scientific investigations of long-term effects of CGF.

## Materials and Methods

### Subjects

This was a cross-sectional study at The First Affiliated Hospital of Soochow University, Suzhou, China. All subjects were divided into exposed and unexposed groups based on whether fetal stages were at the time of CGF (1959–1961). Age related to the CGF was the inclusion criterion. Whole fetal stages must be within the CGF period for the exposed group. For the unexposed group, their fetal stages must be after the CGF but close to the CGF. In this study, smoking (any kind of smoking, such as cigarettes or e-cigarettes, etc.) and drinking alcohol were the factors considered as exclusion criteria. After screening, the number of participants who met theses criterion is 11,417. Those who were born from the end of 1959 to the end of 1961 were defined as exposed population. Since CGF affected whole populations in the whole country, it was hard to find unexposed subjects who were born during 1959–1961. Thus, subjects born after CGF were served in the unexposed group in Set 1. Notably, this caused age differences between the exposed and unexposed groups in Set 1. Thus, Set 2 study used additional years and two steps for preparing data. The first step was collecting data first for the exposed subjects who were born during 1959–1961. The second step was waiting for several years, and then doing related testing and preparing data for the unexposed subjects born after 1961. Thus, the ages in Set 2 were properly balanced between the exposed and unexposed groups.

### Measurements of Weight, Height, Waist Circumference, Body Mass Index, Blood, and Urine Values

Weight, height, and waist circumference (WC) in both the groups were measured by trained nurses or medical professionals. Height and WC were measured to the nearest 0.1 cm, and weight was recorded to the nearest 0.1 kg, while subjects were wearing light clothing without shoes. WC was measured from the distance around the umbilicus horizontally with participants in a standing position. Waist circumference was categorized using WHO cutoff points: abdominal obesity is defined as >102 cm for men and >88 cm for women. Body mass index (BMI, kg/m^2^) was calculated with the formula: weight (kilograms) divided by height (meters squared). General obesity was determined by the BMI, categorized using the cutoff values in China: normal: BMI <25 kg/m^2^, overweight: BMI ≥25 kg/m^2^ and obesity: BMI ≥30 kg/m^2^. The BMI ≥25 kg/m^2^ was defined abnormal (23).

Blood and urine samples were collected in the morning after 8 h of fasting. Plasma samples were frozen at −80°C until testing. Fasting plasma glucose (FPG) was measured using an oxidized enzymatic method. Concentrations of total cholesterol (TC) and triglycerides (TG) were assessed enzymatically using an automatic biochemistry analyzer (Hitachi Inc., Tokyo, Japan) and commercial reagents. Urine sugar (negative and positive) was detected by professional urine sugar dipstick. Fatty liver was detected with B-mode ultra-sonography by the professionals. The normal critical values of blood glucose, TC, and TG were set as 6.1, 5.2, and 1.7 mmol/l, respectively, according to the standards. The data were processed and analyzed in a blind manner.

The main diagnosis of medical conditions is based on the final diagnosis given by doctors, which was also handled in a blind manner to the analysis. The main diagnostic results were divided into two parts, namely, glucose metabolism related dysfunction and lipid metabolism related dysfunction, classified as abnormal and normal, respectively. Those diagnosed with diabetes and increased fasting glucose was classified as abnormal glucose metabolism; those diagnosed with fatty liver, hypercholesterolemia, and elevated triglyceride were classified as abnormal lipid metabolism. In ultrasonography, fatty liver disease was defined by the presence of at least two of three abnormal findings, namely, high hepatorenal echo contrast, sound attenuation, and echo reflectance increased.

### Statistical Analysis

We compared two sets of data according to sexes. Data were presented as mean ± standard deviation (SD) for continuous variables, and percentage for categorical variables. Analyses were conducted using the SPSS 21. *P* < 0.05 was considered statistically significant. Chi-square test and *t*-test were used to compare the exposed group and unexposed group in Set 1 and Set 2, respectively. The data for normal distribution were ensured before analysis. Logistic regression was used to analyze correlation of the risk factor famine exposure to the measured values.

### Ethics

Subjects were given informed consent before examinations by doctors/medical workers regarding their results may be used for scientific analysis. Only those examination results with the agreement of subjects were used for this study. The details of this study were reviewed and approved by the Ethics Committee of The First Affiliated Hospital of Soochow University (ER0201601197).

## Results

### Subject Characteristics

Tables 1, 2 summarize characteristics of the subjects. Comparisons were done between the exposed and unexposed groups with imbalanced ages in Set 1, and the balanced ages in Set 2. There were 6,661 men in Set 1, including 4,298 exposed and 2,363 unexposed, and 4,756 women, with 3,273 exposed and 1,483 unexposed. In Set 2, 5,628 men included 3,265 exposed and 2,363 unexposed, and 4,036 women included 2,553 exposed and 1,483 unexposed.

**TABLE 1:** Physiological and biochemical values for Set 1 (*t*-test).

	Male			Female		
	Exposed (*n* = 4,298)	Unexposed (*n* = 2,363)	*P* value	Exposed (*n* = 3,273)	Unexposed (*n* = 1,483)	*P* value
BMI (kg/m^2^)	25.26 ± 2.73	25.04 ± 2.82	0.017	23.56 ± 3.00	23.67 ± 2.99	0.424
Height (cm)	170.09 ± 6.03	169.81 ± 5.81	0.164	159.09 ± 5.16	159.02 ± 5.31	0.770
Weight (kg)	73.24 ± 9.22	72.52 ± 9.20	0.021	59.43 ± 7.90	59.84 ± 8.01	0.235
Waist circumference (cm)	90.05 ± 7.42	90.19 ± 6.82	0.849	80.45 ± 8.03	78.57 ± 6.96	0.016
Blood glucose (mmol/L)	5.55 ± 1.34	5.58 ± 1.47	0.432	5.30 ± 1.00	5.18 ± 0.90	0.0005
Total cholesterol (mmol/L)	4.90 ± 0.93	4.90 ± 0.89	0.986	5.03 ± 0.85	5.04 ± 0.82	0.587
Triglyceride (mmol/L)	1.97 ± 1.72	1.95 ± 1.62	0.625	1.45 ± 1.13	1.41 ± 0.84	0.199

**TABLE 2:** Physiological and biochemical values for Set 2 (*t*-test).

	Male			Female		
	Exposed (*n* = 3,265)	Unexposed (*n* = 2,363)	*P* value	Exposed (*n* = 2,553)	Unexposed (*n* = 1,483)	*P* value
BMI (kg/m^2^)	25.27 ± 2.74	25.04 ± 2.82	0.019	23.57 ± 2.94	23.67 ± 2.99	0.478
Height (cm)	170.08 ± 6.10	169.81 ± 5.81	0.117	159.32 ± 5.13	159.02 ± 5.31	0.206
Weight (kg)	73.34 ± 9.07	72.52 ± 9.20	0.012	59.61 ± 7.76	59.84 ± 8.01	0.529
Waist circumference (cm)	87.63 ± 7.82	90.19 ± 6.82	0.054	80.20 ± 8.27	78.57 ± 6.96	0.049
Blood glucose (mmol/L)	5.55 ± 1.36	5.58 ± 1.47	0.501	5.24 ± 0.82	5.18 ± 0.90	0.038
Total cholesterol (mmol/L)	4.85 ± 0.90	4.90 ± 0.89	0.065	4.99 ± 0.84	5.04 ± 0.82	0.051
Triglyceride (mmol/L)	2.05 ± 1.83	1.95 ± 1.62	0.039	1.35 ± 0.94	1.41 ± 0.84	0.051

### Comparisons Between the Exposed and Unexposed in Both Men and Women in Set 1

Statistical analysis of BMI and related indexes is summarized in Table 1. The BMI in the exposed men was significantly higher than the unexposed men (*P* < 0.05, 95% CI: 0.04–0.40), while no significant differences were observed in women. In comparison of height, weight, and waist circumference of the exposed and unexposed between sexes, there was no statistical difference in height of the male and female offspring between the exposed and unexposed groups, the weight of the men in the exposed group was significantly higher than that in the unexposed (*P* < 0.05, 95% CI: 0.11–1.31), and there was no significant difference in female weight between the two groups. The mean of WC of women in the exposed group was significantly higher than that of the unexposed (*P* < 0.05, 95% CI: 0.35–3.40), while no statistical differences were observed in men (Table 1).

Table 1 summarizes comparisons of related indexes of glucose and lipid metabolism between the two groups in different sexes. There were no statistical differences in blood glucose, TC, and TG between the two groups in the men, while the blood glucose levels (*P* < 0.001, 95% CI: 0.05–0.18) in the women were significantly higher in the exposed group than those in the unexposed group (Table 1). There was a significant difference in the incidence of lipid metabolic dysfunction in both the men and women of the exposed group compared with the unexposed (*P* < 0.05), although there was no statistical difference in the incidence of glucose metabolic dysfunction between the two groups in either male or female group (Table 3).

**TABLE 3:** Analysis using Pearson’s chi-square test.

	Male (*p* value)	Female (*p* value)
**Set 1**		
Urine sugar	0.179	0.075
Fatty liver	0.119	0.291
Lipid metabolic related disease (principal diagnosis)	0.015	0.044
Glucose metabolic related disease (principal diagnosis)	0.117	0.143
**Set 2**		
Urine sugar	0.409	0.587
Fatty liver	*P* < 0.001	0.382
Lipid metabolic related disease (principal diagnosis)	*P* < 0.001	*P* < 0.001
Glucose metabolic related disease (principal diagnosis)	0.015	*P* < 0.001

Logistic regression analysis in Set 1 is summarized in Table 4, and CGF exposure increased risks of abnormal BMI (*P* < 0.001, OR = 2.641, 95% CI: 2.360–2.955) and blood sugar (*P* < 0.05, OR = 1.181, 95% CI: 1.020–1.356) in the men and abnormal blood sugar (*P* < 0.001, OR = 1.573, 95% CI: 1.241–1.993) and urine sugar (*P* < 0.05, OR = 2.454, 95% CI: 1.017–5.924) in the women.

**TABLE 4:** Logistic regression analysis of famine exposure and glucolipid metabolic indexes.

	Male			Female		
	OR	95% CI		OR	95% CI	
**Set 1**						
BMI	2.641	2.360 ∼ 2.955	*P* < 0.001	1.020	0.844∼ 1.232	–
Waist circumference (WC)	0.366	0.130 ∼ 1.029	–	1.230	0.690∼2.190	*P* < 0.001
Blood Glucose	1.181	1.029 ∼ 1.356	*P* < 0.05	1.573	1.241∼1.993	–
Total cholesterol (TC)	0.981	0.883 ∼ 1.090	–	0.890	0.785 ∼ 1.009	–
Triglyceride (TG)	1.019	0.921 ∼ 1.127	–	1.008	0.875 ∼ 1.160	–
Urine sugar	0.802	0.582 ∼ 1.107	–	2.454	1.017 ∼ 5.924	*P* < 0.05
Fatty liver	1.083	0.979 ∼ 1.199	–	1.079	0.937 ∼ 1.243	–
**Set 2**						
BMI	2.599	2.305 ∼ 2.930	*P* < 0.001	1.023	0.838 ∼ 1.250	–
Waist circumference (WC)	0.401	0.134 ∼ 1.199	–	1.311	0.725 ∼ 2.371	–
Blood glucose	1.213	1.050 ∼ 1.401	*P* < 0.05	1.315	1.024 ∼ 1.689	*P* < 0.05
Total cholesterol (TC)	0.894	0.799 ∼ 1.001	–	0.885	0.776 ∼ 1.008	–
Triglyceride (TG)	1.12	1.006 ∼ 1.245	*P* < 0.05	0.874	0.753 ∼ 1.014	–
Urine sugar	0.867	0.618 ∼ 1.217	–	2.527	1.062 ∼ 6.221	*P* < 0.05
Fatty liver	1.248	1.121 ∼ 1.390	*P* < 0.001	0.936	0.806 ∼ 1.086	–

### Comparisons Between the Exposed and Unexposed in Both Men and Women in Set 2

Table 2 summarizes the independent testing results of the gender-specific exposed and unexposed after excluding age differences. The BMI (*P* < 0.05, 95% CI: 0.04–0.43) and body weight (*P* < 0.05, 95% CI: 0.19–1.46) of the men in the exposed group were significantly higher than that in the unexposed group. There were no statistical differences in BMI, height, and weight between the exposed and unexposed women. Waist circumference analysis showed significant difference in the women but not in the men between the two groups (*P* < 0.05, 95% CI: 0.005–3.25).

As summarized in Table 2, blood glucose (*P* < 0.05, 95% CI: 0.001–0.12) in the exposed women was significantly higher than that in the unexposed women, although no statistical difference was observed in blood glucose for men. TC and TG was not increased in the exposed women compared with the unexposed women, TG was increased significantly in the exposed men compared with the unexposed men (*P* < 0.05, 95% CI: 0.005–0.19), and there was no statistical difference in TC. Table 3 summarizes the results of diagnoses related to glucose and lipid metabolism. The diagnosis of abnormal glucose and lipid metabolism in both male and female exposed group was significantly different from that in the unexposed group (male: abnormal glucose metabolism *P* < 0.001, abnormal lipid metabolism *P* < 0.05; female: abnormal glucose metabolism *P* < 0.001, abnormal lipid metabolism *P* < 0.001). Chi-square analysis showed that there was a statistical difference in fatty liver in the men (*P* < 0.001) but not in the women (Table 3).

Table 4 summarizes the results by logistic regression analysis in Set 2. Exposure to CGF increased risks of abnormal BMI (*P* < 0.001, OR = 2.599, 95% CI: 2.305–2.930), blood sugar (*P* < 0.05, OR = 1.213, 95% CI: 1.050–1.401), and triglycerides (*P* < 0.05, OR = 1.12, 95% CI: 1.006–1.245), and increased risks of fatty liver in the men (*P* < 0.001, OR = 1.248, 95% CI: 1.121–1.39). For women, CGF exposure increased risks of blood sugar (*P* < 0.05, OR = 1.315, 95% CI: 1.024–1.689) and urine sugar (*P* < 0.05, OR = 2.527, 95% CI: 1.062–6.211).

### Comparisons Between Both Sexes in the Exposed or Unexposed Populations

**FIGURE 1:**
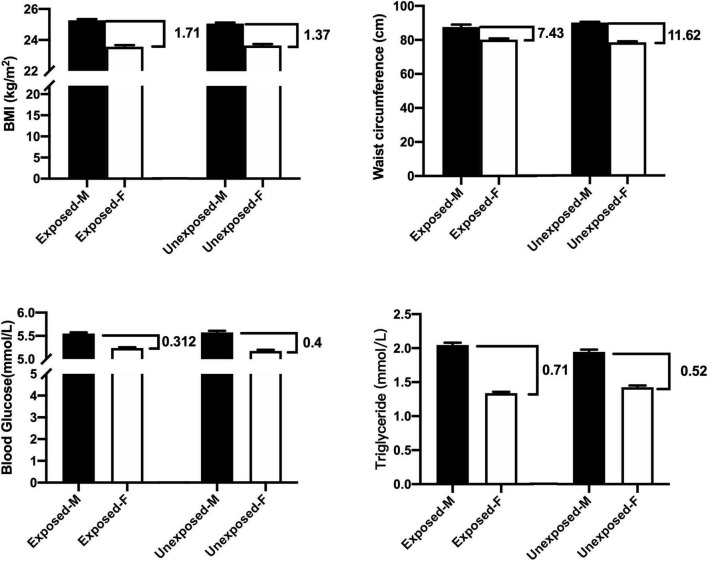
Comparisons between the exposed-M (male) and exposed-F (female) as well as between unexposed-M and unexposed-F (*t*-test).

This study also made comparisons between the exposed men and exposed women as well as between the unexposed men and unexposed women. Figure 1 and Table 5 summarize that the difference in mean of BMI between the men and women was 1.71 in the exposed population and 1.37 in the unexposed population, that is, the difference in BMI between men and women in the exposed population increased by 24.8% compared with that in the unexposed population. Similarly, the mean of WC difference between men and women in the exposed population was 36.8% lower, blood sugar 22% lower, and triglycerides 36.5% higher compared with the unexposed population.

**TABLE 5:** The results in the table were obtained by analysis from Figure 1 with two steps.

	Difference between men and women (Exposed)	Difference between men and women (Unexposed)	Percentage change (%)
BMI (kg/m^2^)	1.71	1.37	24.8 ↑
Waist circumference (cm)	7.34	11.62	36.8 ↓
Blood glucose (mmol/L)	0.312	0.4	22.0 ↓
Triglyceride (mmol/L)	0.71	0.52	36.5 ↑

*Step 1: When the exposed-M (male) was compared to the exposed-F (female) and the unexposed-M compared to the unexposed-F, two differences (Δvalues) were obtained. Step 2: Using Δvalues of the unexposed groups as a base to calculate percentage changes for the exposed group between men and women. Upward arrow presents the increased levels in percentage of men compared to women; downward arrow presents the increased levels in percentage of the women compared to men.*

## Discussion

The first research report of long-term influence of CGF in fetal origins on cardiovascular systems was our study several years ago (24). Since then, a number of studies reported that CGF has a close link to increase incidence of cardiovascular and metabolic diseases (25–28). However, it is noted that CGF affected whole country and population 50 years ago, but the possibility is small to have unexposed subjects as the control with the same ages born during 1959–1961. Thus, most of the studies that used the control as unexposed were subjects born after CGF. This resulted in the exposed subjects in studies being older than the control. Considering aging factors contributing significantly to metabolic and lipid regulations, even differences of several years in the older may cause significant differences in observed indexes between groups. Therefore, this study was designed in comparisons using two sets, i.e., (1) fetal exposure to CGF (1959–1961) vs. non-fetal exposure born after CGF, in which there were age differences between the two groups as being reported by most of the studies on CGF and (2) fetal exposure to CGF (1959–1961) vs. non-fetal exposure born after CGF; however, the subjects in the unexposed group were tested a few years later than the exposed group so that the ages for both groups at testing and analysis were the same and balanced. To the best of our knowledge, this study was the first to balance aging factor in analysis of long-term effects of CGF.

Interestingly, while the numerous results in comparisons between the exposed and unexposed were similar for the two Sets, differences emerged indeed after adjusting aging factors. For example, triglyceride levels in the exposed group were not significantly different from the unexposed group in the Set 1 (ages were not balanced). However, after ages were balanced in the Set 2, the exposed offspring showed increased triglycerides compared with the control. A similar finding was also revealed in comparisons of effects of CGF on fatty liver between the exposed and unexposed in the two Sets. These findings demonstrate that since most of the studies use CGF model recently, all exposed subjects had been at ages over 50 years of age, and aging factor may affect results in comparisons of certain physiological or pathophysiological indexes, even after several years of differences between the groups.

In the theory of “fetal origins of adult diseases,” Barker hypothesized that “something early in life is branded for the rest of a child’s life” (1). That is, poor prenatal factors cause abnormalities in the development of the embryo or fetus, leading to an increased susceptibility to a variety of chronic diseases after birth. As one of the adverse factors during pregnancy, famine has attracted great attention in researching all over the world since Barker’s hypothesis. From the 1994 Dutch famine to the 1959–1961 CGF, poor nutrition affected fetal growth during the famine periods, and its effects impacted significantly on the offspring in metabolism, cardiovascular, or nervous systems in adulthood (24, 29–32). Previous studies demonstrated that the effects of malnutrition on health or diseases could be different between sexes (18, 33–35). Laboratory experiments also showed different influence of malnutrition on cardiovascular or metabolism consequences between men and women (36, 37). However, in most of the previous studies on CGF, very few have looked at possible sex differences in glucose and lipid metabolism in the offspring. This study was the first to focus on that issue in using the CGF model. The information gained will benefit each sex in early prevention and treatments of the diseases in fetal origins. Interestingly, the findings in this study revealed that several critical indexes were different between the male and female offspring following prenatal exposure to CGF. This enriched the Barker’s theory that the same prenatal insult may cause both the same and different consequences between men and women at an early stage of old.

Body mass index, a standard to measure the degree of body fat and fitness, is also closely related to diseases linked to glucose and lipid metabolism. This study compared BMI between sexes in the exposed and unexposed groups. Although not considering Set 1 and Set 2, the male BMI in the exposed group was significantly higher than that in the unexposed group, although there was no statistical difference in women. This result suggests that the male offspring was more important than the female offspring at an early stage of old to prenatal undernutrition. There was no significant difference in height between the two groups for either men or women, and the male weight in the exposed group was significantly higher than that in the unexposed group, although no significant difference in the female weight was observed. Although there were no records of birth weight during CGF that could be obtained, studies on Dutch famine and other models as well as animal studies have demonstrated that a major consequence of famine or poor nutrition is LBW (13, 21, 22). The new findings in this study are very interesting because of the fact that height in the early stage of old was the same between the exposed and the control regardless of different sexes, and their weight was either the same or significantly increased in women and men, which strongly indicates that there exists a “catch-up growth” after birth for both the sexes following prenatal exposure to CGF. This is also the first time to report that “catch-up growth” occurred for the offspring prenatally exposed to CGF.

Body mass index is composed of weight and height; in the case of no differences in height between the two groups, it can be reasonably inferred that the increase in BMI of the men in the exposed group was caused by significantly increased weight. Waist circumference plays an important role in determining both abdominal fat storage and central obesity, and several studies have shown that increased WC is associated with increased risks of cardiovascular and metabolic diseases, even early death (38). In this study, WC of men in the exposed group was not significantly increased compared with the unexposed group. It seems that the weight gained and fat distribution in men may be generalized over the body, instead of central obesity. However, regarding the same ages of women, WC was significantly increased following exposure to CGF.

This study also determined blood glucose and urine glucose related to glucose metabolism, and examined TC and TG, as well as occurrence of fatty liver, reflecting lipid metabolism. Correlation analysis in both Set 1 and Set 2 showed that CGF exposure was a significant risk factor for blood sugar in both men and women. Moreover, urine sugar was also significantly influenced by CGF in the female exposure group, but not in the male exposure group. Although blood cholesterol was not significantly increased following fetal exposure to CGF in both the male and female groups, triglyceride in the men was significantly enhanced in the male exposure group, but not in the women. Furthermore, the occurrence of fatty liver in the men, and not in the women, was significantly related to CGF exposure. Notably, this study was the first to demonstrate a long-term impact of CGF on increased risks of fatty liver with sex differences. This also demonstrated that even after 50 years from birth, prenatal exposure to CGF still could significantly affect lipid metabolisms in the body. Those findings between sexes also raised two questions, i.e., (1) what are the possible mechanisms underlying the long-term influence of CGF on observed abnormalities in metabolisms at early stage of old? and (2) why the influence could be different between sexes?

In term of the mechanisms, “catch-up growth” could be one possibility. Based on solid evidence from numerous studies that poor nutrition or famine could definitely cause fetal growth restriction and low-birth weight (13, 22), and millions died due to food shortage during CGF (14), as well as the offspring height and weight was either the same or higher between the exposed and unexposed, it is reasonable to conclude that “catch-up growth” should occur in the exposed subjects in this study, regardless of sexes. It is known that catch-up growth as an abnormal developmental process can affect metabolisms in the body (19, 20) *via* inducing permanent changes in functions, reducing insulin sensitivity, or increasing circulating insulin levels, leading to insulin resistance (39–41). Another possible underlying mechanism is that epigenetic changes during early developmental periods can produce long-term risks of diseases (42–45). There have been a number of experimental studies that demonstrated DNA methylation induced by malnutrition in early developmental stages could significantly increase risks in cardiovascular and metabolic systems in the offspring (46–49). Malnutrition-induced epigenetic changes could cause weight and fat mass gain, glucose intolerance, hypertriglyceridemia, abnormal adiponectin, and leptin levels in metabolisms in the offspring (50–52). Notably, even there are many studies which prove that the epigenetic mechanisms play critical roles in prenatal insult-induced abnormal metabolic changes in later life, almost all those studies used experimental animal models. Future investigations are expected to determine epigenetic influence on human subjects exposed to CGF during fetal periods.

Why the same famine could cause different consequences between the sexes? In this study, an analysis showed that the CGF exposure significantly increased risks for regulations of BMI, triglyceride, and blood sugar in men at an early stage of old; these critical changes were also associated with higher risks of fatty liver. Regarding the same ages of women, CGF exposure was also a risk factor for the blood sugar. However, analysis did not reveal the same risks as the men in BMI, triglyceride, and fatty liver. It appears that the female offspring might have protective mechanisms in face of long-term challenges from fetal exposure to famine. It is not strange that certain hormonal mechanisms in women can serve in protection of cardiovascular diseases (53–55). Estrogen has been demonstrated repeatedly to affect fat distribution and carbohydrate metabolism against pathophysiological processing in lipid metabolisms (56–58). That is a major reason that risks and incidences of cardiovascular or metabolic diseases are significantly lower in women than men. After menopause, loss of female hormone acts as a privilege against the risks of the diseases (56, 59, 60). Interestingly, new findings in this study demonstrate that the risks of abnormal BMI, triglyceride, and fatty liver still were relatively lower in the women exposed to CGF in fetal stages compared with the men. Those women were around 52–55 years of age who were included in the study, reasonably after menopause for only a few years, supposed to lose privilege of hormone protection. The results in this study suggest that either there may exist additional mechanisms in reducing risk factors from CGF exposure in women, or those subjects were only a few years after menopause; previous influence from hormone privilege has not gone completely. Thus, it is worth noting for further 10-year or longer follow-up studies on those subjects. Another important finding in sex analysis was that besides the privilege, the women also have their weak point. Although fetal exposure to CGF was a significant risk factor to abnormal blood sugar for both the men and women, there was a significant increase of blood sugar levels in the women than that in the men. Moreover, the fetal exposure to CGF was a high risk to urine sugar of women, not to the men. It seems that the women might be more vulnerable than the men in glucose metabolisms in face of long-term challenges from the famine. This Achilles heel in the women and its underlying mechanisms also deserve further investigations. Notably, the findings in this study have revealed both similar and different outcomes between sexes, which provides important information for further investigating and exploring precise medicine or approaches for each sex in the early prevention of metabolic dysfunction and fatty liver.

Table 5 and Figure 1 show differences of means of testing values between the men and women not only as aging factors were balanced, but also with or without famine exposure factors were equal, because the comparisons were performed between the exposed men and exposed women, as well as between the unexposed men and women. These comparisons will create “quantitative figures or information” that may be helpful to view differences in influencing levels of the famine between sexes in quantification. The difference of mean levels of BMI between the exposed men and exposed women was 1.71, while the difference between the unexposed men and women was 1.37. This means that fetal exposure to CGF increased BMI in the male offspring was 24.8% higher than the female offspring at an early old stage. It would be reasonable that 24.8% of the difference indicates quantitatively that men presented such higher levels of risks in quantification for the increased BMI compared with women under the same conditions. Similarly, comparisons showed that CGF-related exposure demonstrated an increase of TG, which was 36.5% higher in the male offspring than the female offspring. However, increased levels of WC as well as blood glucose following CGF exposure was 36.8 and 22% higher in the women than the men at an early stage of old. It is reasonable to assume that the changes in the differences between men and women in the exposed versus unexposed groups could be due to CGF during pregnancy.

## Conclusion

This study proved that undernutrition during pregnancy had a significant long-term impact on metabolic dysfunction in the offspring; such influence could remain for a long time, even 50 years after CGF. Strengths of this study include that this was the first study to identify sex differences in the long-term effects on glycolipid metabolism. This is also the first study to report the presence of a “catch-up growth” after fetal exposure to CGF. Importantly, long-term impact of prenatal malnutrition on metabolic changes and fatty liver could be quite different between sexes, as the new results showed. The importance of the findings of the differences between sexes is that they provide new clues in considering or exploring different strategies of precise medicine for each sex in early prevention of metabolic dysfunction and fatty liver after birth and before aging.

## Data Availability Statement

The data is available *via* the authors upon reasonable requests.

## Ethics Statement

The studies involving human participants were reviewed and approved by the Ethics Committee of The First Affiliated Hospital of Soochow University (ER0201601197). The patients/participants provided their written informed consent to participate in this study.

## Author Contributions

YZ and ZX designed the project and wrote the manuscript. ZX and YG revised the manuscript. LW, YY, ZZ, JP, and DZ collected and sorted data from annual medical examination records. YD performed the statistical analysis. QZ, QH, TX, YH, and HS assisted with data analysis. XZ, LL, and YXY assisted with critical study and figure preparation. All authors contributed to the article and approved the submitted version.

## Conflict of Interest

The authors declare that the research was conducted in the absence of any commercial or financial relationships that could be construed as a potential conflict of interest.

## Publisher’s Note

All claims expressed in this article are solely those of the authors and do not necessarily represent those of their affiliated organizations, or those of the publisher, the editors and the reviewers. Any product that may be evaluated in this article, or claim that may be made by its manufacturer, is not guaranteed or endorsed by the publisher.
